# CD21^+^CD11c^+^ B cells predicting handgrip strength decline in older adults

**DOI:** 10.3389/fragi.2026.1844391

**Published:** 2026-05-29

**Authors:** Diego Mabe-Castro, Matías Mabe-Castro, Lindybeth Sarmiento Varón, Marcelo A. Navarrete, Matías Castillo-Aguilar, Cristian Núñez-Espinosa

**Affiliations:** 1 Escuela de Medicina, Universidad de Magallanes, Punta Arenas, Chile; 2 Centro Asistencial Docente e Investigación (CADI), Universidad de Magallanes, Punta Arenas, Chile

**Keywords:** B-cell, flow cytometry, hand strength, healthy aging, immunosenescence

## Abstract

**Background:**

Preserving physical function is central to healthy aging, and declines in handgrip strength are strong predictors of frailty, disability, and mortality. Although immunosenescence has been implicated in age-related functional decline, the contribution of specific B-cell subsets to longitudinal changes in muscle strength remains poorly understood.

**Objective:**

To examine whether baseline B-cell phenotypes are associated with 1-year changes in handgrip strength in community-dwelling older adults.

**Methods:**

Sixty-two adults aged ≥60 years from the Magallanes Region, Chile, underwent baseline and 1-year follow-up assessments of handgrip strength and body composition. Peripheral B-cell subsets were characterized by flow cytometry, identifying CD45^+^CD19^+^ lymphocytes and classifying subsets according to CD21 and CD11c expression. Associations between baseline B-cell subsets and 1-year change in handgrip strength were evaluated using robust linear regression adjusted for age, sex, and change in muscle mass.

**Results:**

Participants showed a significant 1-year decline in handgrip strength (mean change: −2.02 kg), whereas muscle mass remained stable. Higher baseline frequencies of CD19^+^CD21^+^CD11c^+^ B cells were independently associated with greater decline in handgrip strength over 12 months (βstd = −0.31, CI95% [-0.60, −0.03], p = 0.031). No other B-cell subset was associated with changes in handgrip strength or muscle mass.

**Conclusion:**

Baseline levels of CD19^+^CD21^+^CD11c^+^ B cells were associated with a subsequent decline in handgrip strength in community-dwelling older adults, independent of change in muscle mass. These findings support a potential link between B-cell phenotypes and functional decline, warranting further investigation in larger cohorts.

## 1 Introduction

With the global population continuing to age, maintaining physical function and independence among older adults has become a critical public health priority (Beard et al., 2016). Among functional measures, handgrip strength (HGS) has emerged as a simple, reliable, and clinically meaningful measure of physical function and a robust predictor of adverse outcomes in older adults, including frailty, disability, cognitive decline, and mortality (Celis-Morales et al., 2018; Cui et al., 2021; Peterson et al., 2023; Andersen et al., 2024). Notably, declines in grip strength often exceed losses in muscle mass, suggesting that neuromuscular integrity and systemic regulatory homeostasis (rather than mere tissue quantity) play a role in functional aging (Auyeung et al., 2014; Wiegmann et al., 2021).

Increasing evidence suggests that age-related immune remodeling may contribute to the neuromuscular mechanisms underlying this functional decline (Li and Ma, 2024; Nguyen and Cho, 2025). Immunosenescence and inflammaging (the chronic low-grade inflammatory state associated with aging) have been implicated in multiple age-related conditions, including frailty, disability, and most non-communicable chronic diseases (Ajoolabady et al., 2024). These conditions are characterized by persistent immune activation and accumulation of senescent cells (Andonian et al., 2025). These cells exhibit Senescence-Associated Secretory Phenotype (SASP), secreting proinflammatory mediators that can impair multisystemic function (Gong et al., 2025).

Among these immune changes, unconventional B-cell subsets accumulate with aging and chronic inflammation (Gjertsson et al., 2022; Lee et al., 2022). These populations, often referred to as age-associated B cells (ABCs), are classically characterized by CD11c expression together with absent CD21 expression (CD21^−^), a phenotype linked to a more proinflammatory functional state, including enhanced cytokine production (Jenks et al., 2018; Golinski et al., 2020; Reincke et al., 2020; Gjertsson et al., 2022; Lee et al., 2022). ABC-like B-cell populations have been described in advanced immune aging and autoimmunity (Claes et al., 2016; Cancro, 2020; Rincón-Arevalo et al., 2021). However, the CD21^low/+^CD11c^+^ B-cell phenotype may reflect an earlier immune state, associated with reduced homeostatic resilience and potentially representing a transitional stage preceding overt senescence.

Recent research from our group has established a link between these specific B-cell phenotypes and reduced physiological resilience, where higher frequencies of CD21^+^CD11c^+^ B cells are associated with impaired cardiac autonomic signatures and slower vagal recovery following physical stress (Castillo-Aguilar et al., 2026a; Castillo-Aguilar et al., 2026b). These findings suggest that immune-driven autonomic dysregulation may be a precursor to broad functional failure; however, the role of these specific immune populations in predicting long-term reductions in physical strength remains incompletely understood.

In this context, recent transcriptomic evidence identifies CD21^+^ B cells as a distinct lineage of activated precursors primed for effector functions (Keller et al., 2021; Yang et al., 2022; Keller and Warnatz, 2023), suggesting they may represent a more proximal precursor to functional decline than terminal ABCs. In this longitudinal cohort of community-dwelling older adults, we hypothesized that the baseline frequency of CD19^+^CD21^+^CD11c^+^ B cells predicts a 1-year decline in handgrip strength, potentially occurring independently of changes in total muscle mass, thus identifying an early immunological signature of dynapenia.

Therefore, this study aims to quantify the mean annual change in grip strength and evaluate its association with baseline B-cell phenotypes. By linking immune-regulatory signatures to long-term functional trajectories, our work contributes to the understanding of the neuro-immune-muscle axis in aging, offering insights into early biomarkers for interventions aimed at preserving healthspan.

## 2 Materials and methods

### 2.1 Study design

A 1-year longitudinal observational study was conducted to assess the temporal change in handgrip strength and B-cell phenotypes in community-dwelling older adults. All measurements were performed at the Centro Asistencial Docente e Investigación de la Universidad de Magallanes (CADI-UMAG), Punta Arenas, Chile. Data were collected longitudinally on two occasions, 12 months apart, during March and April of consecutive years. To minimize diurnal variation, all assessments were conducted between 08:00 and 11:00 a.m. under fasting conditions.

### 2.2 Participants

Sixty-two community-dwelling older adults were recruited through non-probability convenience sampling. Inclusion criteria were: a) permanent residency in the Magallanes and Chilean Antarctic Region, and b) age ≥60 years. Exclusion criteria included: a) neurological or musculoskeletal conditions affecting hand function, b) cognitive impairment the ability to follow instructions, c) diagnosed autoimmune disease, d) active cancer of history of chemotherapy within the last 12 months, e) use of immunosupressive or corticosteroid medication within the past 6 months, f) acute infections at the time of sampling, and g) major surgery or receiving blood transfusions within the previous 3 months.

The protocol was approved by the Scientific Ethics Committee of the University of Magallanes (No. 001/SH/2025) and conducted in accordance with the Declaration of Helsinki on ethical principles for medical research. All participants provided written informed consent prior to enrollment.

### 2.3 Procedures and instruments

#### 2.3.1 Handgrip strength assessment

HGS was measured using an electronic hand dynamometer (CAMRY EH101, Sensun Weighing Apparatus Group Ltd, Guangdong, China), which has demonstrated high validity and reliability in older populations (Huang et al., 2022; Sánchez-Aranda et al., 2025) and provides measurements with a precision of 0.1 Kg. Following the American Society of Hand Therapists (ASHT) recommendations, participants were seated with the shoulder adducted and neutrally rotated, the elbow flexed at 90°, and the forearm and wrist in a neutral position (MacDermid et al., 2015). After adjusting the handle to the participant’s hand size, they were instructed to exert maximal isometric strength for at least 3 s. Three trials were performed for each hand with 1-min rest intervals. The primary outcome was the mean of the maximum values from both hands (kg). The same chair, position, and instructions were given for both evaluation moments.

#### 2.3.2 Peripheral blood collection and B- cell phenotyping

Peripheral blood samples were collected in EDTA tubes and processed within 2 h. To characterize B cell subpopulations, we used fluorochrome-conjugated monoclonal antibodies (mAbs) specific for human CD45 (Brilliant Violet 570, clone HI30, CD19 (FITC, clone HIB19), CD21 (APC A750, clone Bu32), and CD11c (PB 450, clone 3.9). Viability was assessed using 7-AAD (PC5.5). All reagents were from BioLegend (San Diego, CA, United States). For each assay, 100 μL of whole blood (approximately 1 × 10^6^ cells) was incubated with the antibody cocktail for 30 min at room temperature in the dark. After staining, red blood cells were lysed by adding 125 μL of OptiLyse C (Beckman Coulter), followed by brief mixing and a 10-min incubation in the dark. Cells were then washed with 1 mL of IsoFlow™ buffer (Beckman Coulter) to remove debris and unbound antibodies.

**FIGURE 1:**
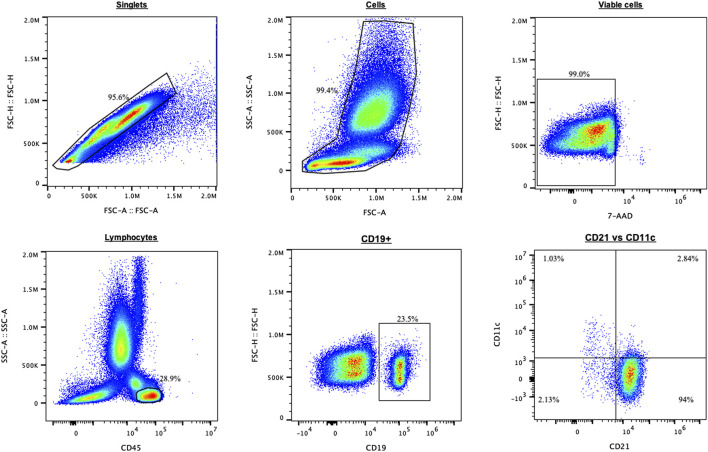
Representative sequential gating strategy used to identify viable CD19^+^ B cells and their distribution according to CD21 and CD11c expression. Gates were sequentially applied to singlets, cells, viable cells, lymphocytes, and CD19^+^ cells. A minimum of 100,000 CD19^+^ events were acquired per sample. The upper-right quadrant corresponds to CD19^+^CD21^+^CD11c^+^ cells.

Samples were acquired on a CytoFLEX S flow cytometer (Beckman, CA, United States), and analyzed using FlowJo software (Tree Star, OR, United States). A minimum of 100,000 CD19^+^ events were acquired per sample. Fluorescence compensation was performed using single-stained compensation beads (BioLegend). Fluorescence minus one and isotype-matched Ab controls were used to set analysis gates. The gating strategy (Figure 1) followed a sequential selection of CD45^+^ lymphocytes, exclusion of 7-AAD^+^ dead cells, and identification of CD19^+^ B cells. In line with our previous work (Castillo-Aguilar et al., 2026a), and following the standardized nomenclature for unconventional B cells in aging and chronic inflammation (Wehr et al., 2004; Keller and Warnatz, 2023), we defined subsets based on the differential expression of CD21 and CD11c. The primary B cell phenotype of interest was the CD21^+^CD11c^+^, whereas the CD21^low/−^CD11c^+^ subset was considered an ABC-like B-cell population (Pinto et al., 2023; McGrath et al., 2024).

#### 2.3.3 Body composition

Bioelectrical impedance analysis (BIA) was performed using a multi-frequency Tanita BC-558 Ironman Segmental Body Composition Monitor (Tanita Ironman, Arlington Heights, IL, United States), with a concordance of 89.3% compared to the Dual X-ray Absorption test using standard measurement protocols (Mialich et al., 2011). Total and segmental muscle mass (kg) and body fat percentage (%) were recorded. Participants followed standard pre-assessment protocols: a 4-h fast, no strenuous exercise or alcohol for 12 h, and bladder voiding prior to measurement.

### 2.4 Statistical analysis

Descriptive statistics (mean ± SD or n, %) were used to characterize the cohort. Spearman’s rho (*ρ*) was utilized to assess baseline correlations between immunological, functional, and morphological variables. To estimate the association between baseline B-cell subset frequencies and 12-month change in HGS, we fitted robust linear regression models (Iterative Reweighted Least Squares, IRLS). This approach was chosen to minimize the influence of outliers and provide more reliable estimates in longitudinal physiological data. Models were fitted, adjusting for sex, age, and the 1-year change in relative muscle mass. All predictors were standardized (z-scores) to allow for direct comparison of effect sizes (βstd). Statistical significance was set at p < 0.05. Analyses were performed in R v4.4.1 using the robustbase and parameters packages, and RStudio graphical user interface (v2024.12.0.467).

## 3 Results

### 3.1 Sample characterization

The final longitudinal cohort consisted of 62 older adults (19 men and 43 women). The participants’ ages ranged from 62 to 89 years, with a median age of 71.5 years (mean ± SD: 71.3 ± 6.2). Baseline descriptive statistics, including body composition, functional parameters, and B-cell immunophenotyping, are detailed in Table 1.

**TABLE 1:** Baseline demographic, clinical, and immunological characteristics of the study cohort stratified by sex.

Characteristic	Overall (n = 62)	Male (n = 19)	Female (n = 43)	SMD^a^	95% CI^b^
Age (years old)	71.3 ± 6.2	73.5 ± 6.0	70.3 ± 6.2	0.54	0.00, 1.1
Weight (kg)	73.8 ± 14.1	76.6 ± 10.4	72.5 ± 15.4	0.32	−0.23, 0.86
Height (cm)	157.5 ± 9.5	165.2 ± 7.7	154.2 ± 8.2	1.4	0.82, 2.0
BMI (kg/m^2^)	29.8 ± 5.6	28.1 ± 3.7	30.6 ± 6.2	−0.49	−1.0, 0.06
Muscle mass (%)	61.80 ± 8.88	72.31 ± 5.57	57.04 ± 5.22	2.9	2.1, 3.6
Body fat (%)	34.8 ± 9.1	24.2 ± 6.4	39.6 ± 5.4	−2.7	−3.4, −1.9
Right handgrip strength (kg)	26.1 ± 7.5	34.3 ± 5.2	22.3 ± 4.8	2.5	1.7, 3.2
Left handgrip strength (kg)	24.5 ± 7.1	31.5 ± 6.2	21.2 ± 4.7	1.9	1.3, 2.6
Total lymphocytes (%)	29.9 ± 9.3	28.4 ± 8.4	30.7 ± 9.9	−0.26	−0.96, 0.45
B lymphocytes (%)	12.4 ± 4.9	9.5 ± 3.6	14.0 ± 4.9	−1.1	−1.8, −0.32
CD19^+^CD21^−^CD11c^+^ (%)	4.1 ± 2.8	3.8 ± 2.7	4.3 ± 3.0	−0.21	−1.6, 1.1
CD19^+^CD21^+^CD11c^+^ (%)	3.7 ± 3.2	0.8 ± 0.5	5.0 ± 3.0	−2.1	−3.7, −0.45
CD19^+^CD21^+^CD11c^−^ (%)	77.4 ± 17.7	70.5 ± 32.0	80.4 ± 9.7	−0.51	−1.9, 0.86
CD19^+^CD21^−^CD11c^−^ (%)	14.8 ± 17.1	25.0 ± 32.1	10.4 ± 4.8	0.78	−0.62, 2.2

^a^ SMD: standardized mean difference, SMD >0.8 indicates a large effect size.

^b^ b. 95% CI: 95% Confidence Interval for the SMD., Continuous variables are expressed as Mean ± SD; Categorical variables as “n” (%).

Data are presented as mean ± standard deviation (SD) for continuous variables and absolute and relative frequencies n (%) for categorical variables. Standardized mean differences (SMDs) and 95% confidence intervals (95% CIs) present the magnitude of the difference between male and female participants.

Significant sex-based differences were observed in body composition and physical performance. At baseline, males exhibited a substantially higher absolute muscle mass compared to females (55.1 ± 6.6 kg vs. 40.4 ± 5.7 kg; SMD = 2.6), consistent with their significantly greater handgrip strength (HGS) for both the right (34.3 ± 5.2 kg vs. 22.3 ± 4.8 kg; SMD = 2.5) and left hand (31.5 ± 6.2 kg vs. 21.2 ± 4.7 kg; SMD = 1.9). Conversely, females presented a higher body fat percentage (39.6% ± 5.4% vs. 24.2% ± 6.4%; SMD = - 2.7).

Regarding the immunological profile, a striking sex-specific difference was identified in B-cell activation markers. While the total B-lymphocyte percentage was higher in females (14.0% ± 4.9% vs. 9.5% ± 3.6%; SMD = −1.1), the most pronounced difference occurred in the CD19^+^CD21^+^CD11c^+^ subset. Female participants exhibited more than six times the relative frequency of this phenotype compared to males (5.0% ± 3.0% vs. 0.8% ± 0.5%; SMD = −2.1). This identifies a baseline immunological signature that, despite the initial differences in muscle mass, serves as a critical predictor for future functional decline.

### 3.2 Baseline associations and temporal dynamics

When examining baseline interactions between systemic biological variables and functional assessments, clear associations emerged. We observed a positive and significant correlation between muscle mass and HGS (*ρ* = 0.63, CI_95%_ [0.42, 0.77], p < 0.001), suggesting that greater muscle mass is associated with better initial strength. Conversely, HGS showed an inverse correlation with fat mass (*ρ* = −0.42, CI_95%_ [-0.62, −0.16], p = 0.002). Furthermore, the analysis of these gross measures with cellular ones revealed an inverse relationship between the relative proportions of B-cells and HGS (*ρ* = −0.44, CI_95%_ [-0.69, −0.11], p = 0.009). Despite these cross-sectional links, no significant associations were found between baseline morphological or functional variables, including muscle mass, and the subsequent temporal change in strength.

When inspecting temporal changes over a 1-year follow-up, a significant decrease in strength was observed (β_std_ = −0.22, CI_95%_ [-0.44, 0.00], p = 0.049). However, muscle mass did not change significantly during this period (β_std_ = 0.03, CI_95%_ [-0.05, 0.10], p = 0.490), indicating that the observed decline is related to neuromuscular function rather than structural atrophy in this cohort.

**FIGURE 2:**
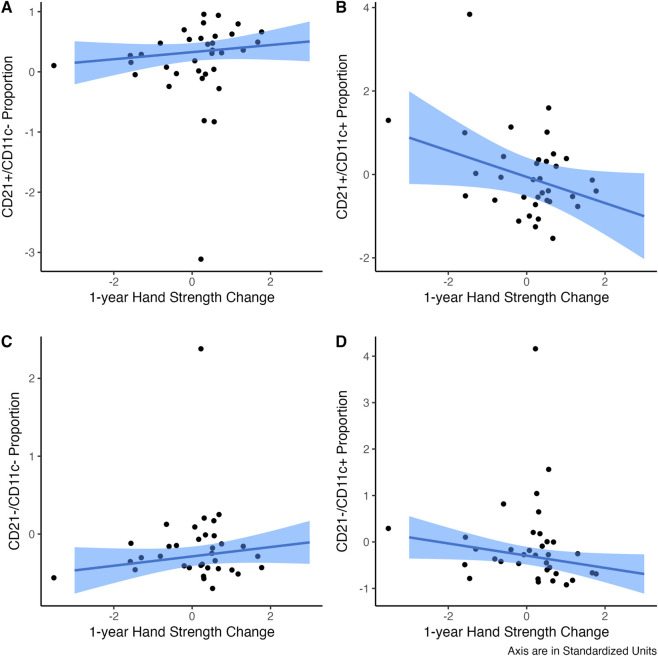
Observed (black dots) and predicted (blue line) with 95% confidence intervals (shaded area) from adjusted models indicating the relationship between different B-cell phenotypes: **(A)** CD21^+^CD11c^−^, **(B)** CD21^+^CD11c^+^, **(C)** CD21^−^CD11c^−^, **(D)** CD21^−^CD11c^+^; and 1-year change in handgrip strength. Model estimates are adjusted for outliers and confounders. All units are in standardized units.

Importantly, when assessing the effect of specific immune phenotypes on these functional changes (Figure 2), we observed that baseline levels of CD21^+^CD11c^+^ B cells were associated with marked decreases in HGS (β_std_ = −0.31, CI_95%_ [-0.60, −0.03], p = 0.031). This association remained statistically significant after adjustment for age, sex, and change in muscle mass. This suggests that the CD21^+^CD11c^+^ signature captures a unique biological process of aging that is not merely a proxy for chronological age. Notably, these same B cell subpopulation levels were not associated with changes in muscle mass (β_std_ = −0.07, CI_95%_ [-0.35, 0.21], p = 0.611), and no other immune cell phenotypes were associated with 1-year functional or morphological changes. These findings support an association between the CD21^+^CD11c^+^ B-cell phenotype and the dissociation between strength decline and stable muscle mass observed over follow-up.

## 4 Discussion

This prospective, community-based cohort explored the longitudinal association between four B-cell immunophenotypes defined by CD21 and CD11c expression and 1-year change in physical functions. Our results demonstrate a significant annual decline in HGS (∼2.0 kg) despite stable total muscle mass. Crucially, the CD21^+^CD11c^+^ subset emerged as a robust immunological predictor of this strength decline, independent of body composition and baseline covariates. These findings suggest that higher baseline CD21^+^CD11c^+^ B-cell levels are associated with subsequent strength decline despite stable muscle mass, supporting further evaluation of this phenotype as a correlate of neuromuscular vulnerability in aging.

### 4.1 The dynapenia phenotype: functional vs. structural aging

The average HGS decline in our cohort exceeds rates typically reported in younger-old populations, approaching the decline observed in very old cohorts (i.e., >85 years) (O’Keefe et al., 2022; Granic et al., 2016). Given that our use of standardized procedures, this suggests a meaningful biological shift rather than measurement variability (Auyeung et al., 2014; Sousa-Santos and Amaral, 2017; McGrath et al., 2022). Together, these data position our cohort at the steeper end of the expected range for community-dwelling older adults and underscore the clinical salience of the immunologic associations detected.

Importantly, the observed decline in strength occurred without significant changes in total muscle mass, a hallmark of dynapenia (Clark and Manini, 2008). This dissociation suggests that, in community-dwelling older adults, age-related functional decline may not entirely be explained by simple atrophy, but rather by deterioration in muscle quality and neuromuscular integrity. Mechanisms such as motor-unit remodeling, altered denervation-reinnervation dynamics, neuromuscular junction dysfunctions, impaired excitation-contraction coupling, and increased fibro-adipogenesis are likely involved (Hunter et al., 2016; Piasecki et al., 2016; Jones et al., 2022; Arnold and Clark, 2023; Brorson et al., 2025). Our findings suggests that immunological aging may target these functional processes before structural sarcopenia becomes evident.

### 4.2 The CD21^+^CD11c^+^ signature and the neuro-immune-muscle axis

The observed dissociation between strength and mass in our cohort characterizes the dynapenia phenotype, where functional loss is driven by the deterioration of neuromuscular quality rather than simple tissue atrophy (Clark and Manini, 2008). Our findings identify a specific immune correlate for this process. While CD21^low/−^CD11c^+^ populations have often been associated with advanced senescence, the CD21^+^CD11c^+^ subset identified here may reflect a distinct activated B-cell phenotype (Ferrucci and Fabbri, 2018; Alberro et al., 2021; Abdullah et al., 2024; Pan and Ma, 2024).

Advanced single-cell transcriptomic profiling has recently identified CD21^+^ B cells as a distinct lineage of activated precursors that expand under chronic inflammatory conditions (Yang et al., 2022). These cells are characterized by high expression of *ITGAX* (CD11c) and a transcriptomic signature 'primed’ for proinflammatory effector functions. Our longitudinal data align with this paradigm, suggesting that the enrichment of the CD19^+^CD21^+^CD11c^+^ phenotype may represent a state of heightened immune surveillance and metabolic activation. This phenotype may be consistent with inflammatory states associated with aging and may help explain why strength decline can occur in the absence of major changes in muscle mass (Keller et al., 2021; Keller and Warnatz, 2023).

The relevance of this phenotype is further supported by our recent work on physiological resilience. We previously demonstrated that these same B-cell profiles are part of a Cardiac Autonomic Signature (CAS), where baseline enrichment of CD11c^+^ and CD21^+^ B cells predicts impaired vagal recovery and altered heart rate variability following exercise (Castillo-Aguilar et al., 2026a; Castillo-Aguilar et al., 2026b). Integrating these findings, we suggest a neuro-immune-muscle framework linking immune activation with autonomic and functional alterations, where the CD21^+^CD11c^+^ phenotype acts as a peripheral marker of a chronic inflammatory state that affects both autonomic regulation and neuromuscular efficiency.

In this context, autonomic nervous system dysfunction contributes to inflammaging (Giunta et al., 2024; Olivieri et al., 2024), and proinflammatory cytokines secreted by these “primed” B cells may disrupt the inflammatory reflex (Tracey, 2002). This disruption could alter neural drive and motor unit recruitment or directly affect motor neurons and the neuromuscular junction (NMJ) integrity long before structural sarcopenia becomes evident. Altogether, our results suggest that enrichment of the CD21^+^CD11c^+^ B-cell population phenotype may reflect a state associated with reduced multi-system resilience in older adults.

Conversely, it cannot be excluded that changes in muscle function, or upstream determinants such as lifestyle habits, general health status, or comorbidity burden, may themselves influence B-cell distribution. Skeletal muscle acts as an endocrine organ, releasing myokines capable of modulating immune function, and reduced muscle activity has been associated with systemic inflammatory alterations (Bruunsgaard, 2005; Iglesias, 2025; Wang et al., 2025). This raises the possibility that the observed immune signatures may partially reflect downstream adaptations to functional decline rather than a unidirectional causal driver.

Epidemiological and basic studies have primarily linked elevated inflammatory markers (such as IL-6 and CRP) with reduced muscle function and accelerated functional decline, supporting the role of inflammation in the decrease of muscular function with aging (Sciorati et al., 2020; Abdullah et al., 2024; Pan and Ma, 2024). While our results resonate with this literature, they should be interpreted with caution, given their observational nature and the absence of mechanistic assays. These findings remain hypothesis-generating but underscore the need for further research integrating functional assays for B-cell subsets and testing their potential utility as aging and functional biomarkers.

### 4.3 Sex-based differences and immune burden

Our analysis revealed a striking sexual dimorphism, with females exhibiting a six-fold higher frequency of the CD21^+^CD11c^+^ phenotype compared to males. Despite these baseline differences, the B-cell signature remained a significant predictor of strength decline even after adjusting for sex. This marked sex difference suggests potential biological heterogeneity that warrants dedicated sex-stratified analyses in future studies.

## 5 Limitations

This study should be interpreted in light of several considerations. The modest sample size and cohort composition constrain statistical power for subgroup analyses and limits more detailed comparisons, particularly in sex-stratified modeling. In addition, B-cell subsets were defined using CD21 and CD11c only, without additional markers for deeper phenotypic resolution. Finally, because the study was observational, the findings support association rather than causation and warrant confirmation in larger and mechanistic cohorts.

## 6 Conclusion

This cohort of community-dwelling older adults exhibited a significant decline in handgrip strength in 12 months, without concomitant changes in muscle mass. Higher basal frequency of CD19^+^CD21^+^CD11c^+^ B cells was associated with greater strength decline over time, with females showing higher levels of total B lymphocytes and CD19^+^CD21^+^CD11c^+^ B cells compared to males. These results support further evaluation of this phenotype as a potential marker of early functional vulnerability in aging and reinforce the relevance of neuro-immune interactions in functional decline.

## Data Availability

The raw data supporting the conclusions of this article will be made available by the authors, without undue reservation.
